# Effects of Selenium in the MAPK Signaling Cascade

**DOI:** 10.15171/jcvtr.2015.23

**Published:** 2015

**Authors:** Nadereh Rashtchizadeh, Pouran Karimi, Parvin Dehgan, Mohamadreza Salimi Movahed

**Affiliations:** ^1^ Drug Applied Research Center, Tabriz University of Medical Sciences, Tabriz, Iran; ^2^ Neurosciences Research Center, Tabriz University of Medical Sciences, Tabriz, Iran; ^3^ Faculty of Nutrition, Tabriz University of Medical Sciences, Tabriz, Iran; ^4^ Faculty of Dentistry, Tabriz University of Medical Sciences, Tabriz, Iran

**Keywords:** Atherosclerosis, Mitogen-Activated Protein Kinase, Platelets, Selenium

## Abstract

*Introduction:* This study aimed to discover by which mechanism selenium (Se) suppresses stimulated platelets stimulation in oxidative stress underlying diseases.

*Methods:* Human platelets pretreated with Se and stimulated by Cu^2+^-oxidized low density of lipoprotein (OxLDL) or thrombin before assessment of P-selectin and phosphorylated p38 mitogen-activated protein kinase (p-p38MAPK), phosphorylated Jun N-terminal kinase (p– JNK), and phosphorylated extracellular signal-regulated kinases (p-ERK1/2). All variables were measured by solid phase sandwich enzyme-linked immunosorbent assay (ELISA).

*Results:* Se significantly decreased Cu^2+^-OxLDL induced P-selectin expression, as well as p38 and JNK phosphorylation in platelets, but could not significantly reduce ERK1/2 phosphorylation.

*Conclusion:* Se suppresses inflamed platelets. This effect maybe partly mediated by the p38 or c-JNK signaling pathways. These results create possibility of new co-anti-inflammatory insight for Se in atherosclerosis.

## Introduction

Platelets as inflammatory cells have a crucial role in the pathophysiology of atherosclerosis.^1^ Stimulation of scavenger receptors on the surface of platelets by oxidative agents, such as oxidized LDL (OxLDL), induces phosphorylation cascades^2^ that leads to platelet activation and translocation of adhesive molecules such as P-selectin from dense granules to the platelets surface and following platelet-dependent thrombosis if continued.

Accumulative evidence have shown that the mitogen-activated protein kinase (MAPK) family is mainly involved in the inflammation and coagulation^3^ JNK, ERK1/2, and p38 are 3 important members of the MAPK family.^4^ The MAPK pathway kinases are triggered when platelets are exposed to extracellular stimuli, including oxidative stress, osmotic shock, inflammatory cytokines, growth factors, and thrombotic agents.^5^

Two potent stimuli among them are OxLDL and thrombin which has been extensively studied.^2,3,6^ Activation by oxidative versus thrombotic agents has exhibited distinctive differences in receptors, signal transduction, and even in upstream activated enzymes.^7^ Thrombin binds to protease activated receptors 1 and 4 (PPAR 1, 4) on the platelet surface that followed by the activation of p38MAPK-NF-κB signaling.^8^ However, based on the anucleated nature of platelets, the involvement of a nuclear factor would be considerable. The OxLDL triggers the same pathway using different receptors, such as toll-like receptors (TLRs) or LOX-1,^9,10^ as well as different mediators that remains unclear yet.

Selenium (Se) is a main ingredient of the glutathione peroxidase that protects cells from the oxidative damage resulting from reactive oxygen species (ROS).^11^ Many studies have indicated the benefits that Se may have on patients with hypertension^12^ and atherothrombotic diseases.^13-17^ Based on the involvement of platelets in these pathologic events, in current study we focused on circulating platelets. Few studies have investigated the mechanism of effect of Se on platelets, despite the common assessments of Se and selenoproteins in different circumstances.^12,14^ We assumed that Se has a role in redox state, altering the activity of the upstream kinases/phosphatases, which in turn may control the phosphorylation status of the MAPK pathways. Hence, this study planned to explore whether or not Se can relieve OxLDL induced inflammation in platelets, as well as the mechanisms by which affects on anti-inflammatory process. So we tested the human platelets exposed to thrombin or Cu^+2^-OxLDL, following phosphorylation status of JNK, p38, and ERK1/2 and finally P-selectin expression as an indicator of thrombus formation. These basic knowledge will be able us to know possibility of suppress of platelets by Se via inhibition of MAPK signaling pathway. We used the pharmacologic inhibitors of MAPKs in order to simultaneously compare with the Se effects.

## Material and Methods

PD98059 (PD), SB203580 (SB) and SP6000125 (SP) were purchased from Calbiochem (San Diego, CA, USA). All enzyme-linked immunosorbent assay (ELISA) kits for phospho-p38 MAPK (Product No: CS0020), total p38 MAPK (Product No: PM0100), Phospho-JNK1&2 (Product No: CS0130), JNK 1&2 (Product No: CS01000), Phospho-ERK1/2 (Product No: CS 7177), total ERK1/2 (Product No: CS 7050), and P-selectin ELISA kit (Catalog Number RAB0426.) were from Sigma Aldrich.

### Preparation of Platelet Samples

Whole blood samples were withdrawn from 10 healthy Tabriz volunteers (aged 45±10 years) who had not received any medical services for the previous 30 days. All volunteers agreed to participate in the study. Samples were obtained based on the Ethical Committee of the Tabriz University of Medical Sciences. The samples with sP-selectin>40 ng/ml as an indicator of platelet activation were excluded. The blood samples were collected in ACD (8.1:1.9 [v/v] 65 mM citric acid and 85 mM citrate plus 111 mM glucose) then centrifuged at 200 g for 8 minutes to prepare the pooled platelet-rich plasma (PRP). The PRP was centrifuged (Beckman Coulter Allegra X-22R Centrifuge Brea, CA, USA) at 8000 g for 10 minutes at 4°C. The platelet pellet was then suspended in a Tyrode’s buffer (140 mM NaCl, 3 mM KCl, 12 mM NaHCO_3_, 0.4 mM _NaH2PO4_, 1 mM MgCl_2_, 2 mM CaCl_2_, 5.6 mM glucose, pH 7.4) to a final concentration of 5×10^8^ platelets/ml.^11^ The platelet count in PRP was estimated by Beckman Coulter (Brea, CA, USA). The means of the platelet counts in any of the groups were not statistically different from each other (*P* =.548).

### Preparation of OxLDL

EDTA was eliminated from commercial LDL by dialyzing against PBS (10 mM NaH_2_PO_4_, 120 mM NaCl, 2.7 mM KCl, and pH 7.4). The LDLs were then oxidized (1 mg/ml) using10 μM CuSO_4_ in PBS overnight at 37°C. Malondialdehyde was measured as a lipid peroxidation index^4^ by thiobarbituric assay.

The study was carried out on 3 groups of platelets: control or resting platelet (RP), 50 µg/ml Cu^2+^-OxLDL treated platelets (OP), and 0.5 U/ml thrombin-activated platelets (TP). Treatment by stimuli was conducted for 10 minutes. Platelet aggregation was monitored on an aggregometer (Multiplate 5.0 analyzer, Dynabyte Medical GmbH). Both OP and TP were considered in both the presence and absence of 100 nmol/lSe for 48 hours to elucidate the effects of Se on P-selectin expression and phosphorylation status of p38, ERK1/2, and JNK. Similarly, 5 × 10^3^ nmol/l SB203580, 400 nmol/lJNK inhibitor SP6000125, or10 µM ERK kinase inhibitor PD980559 were used.

### Preparation of Platelets Lysate

The treated platelets were hemogenized within lysis buffer containing 10 mM Tris pH 7.4, 100 mM NaCl, 2 mM EDTA, TritonX-100, 10% Glycerol, 2 mM Na_3_VO, 0.1% SDS 20 mM Na_4_P_2_O_7_, 1 mM NaF, 0.5% deoxycholate, 1 mM PMSF before centrifugation at 13000 rpm at 4°C for 10 minutes to eliminate the debris. The supernatant was subjected to solid phase sandwich ELISA in a similar manner.

### Enzyme Linked-Immuno-Sorbent Assay (ELISA)

The ELISA procedure is briefly described as follows. All experiments were done in duplicate. After equilibration of the contents of the kits to room temperature, 100 μl standard dilutions, control specimens, and unknown samples (diluted 1:10) were pipetted into specific antibody coated wells and incubated for at least 2 hours at room temperature (RT). Then incubation with 100 μl of one of the primary antibodies (anti-phospho-ERK1/2 [pThr202/Tyr204]), anti-ERK1/2 anti-phosphop 38 (pThr180/pTyr182), anti-p38, anti-phospho-JNK (pThr183/pTyr185), or anti-JNK for 1 hour at RT was carried out. The plates were undergone 3 times washing with PBS-T and incubation with blocking buffer containing anti-Rabbit IgG-HRP conjugated antibody for 30 minutes at RT. After washing 3 times washes with PBS-T, the plates were incubated with stabilized chromogen for 30 minutes at RT. The ELISA reaction was halted by the addition of 1M H_2_SO_4_, and the signals were measured by a spectrophotometer (VersaMax^TM^ Absorbance Micro plate Reader, Molecular Devices, LLC, US) at 450 nm, with plate background correction at 540 nm. Platelet P-selectin was measured by ELISA in the same manner.

### Statistical Analyses

Statistical analyses were conducted using SPSS software (Version13.0; SPSS, Chicago, IL, USA), as well as Mann-Whitney U test and Kruskal-Wallis H test. *P*<.05 was considered significant. The results were shown by the ratio percentage of the phosphorylated to the total proteins for normalizing the phospho-protein contents of the cells (except for P-selectin, which was presented as mean±SD).

## Results

### Inhibition of p38 Phosphorylation by Selenium

**Figure 1:**
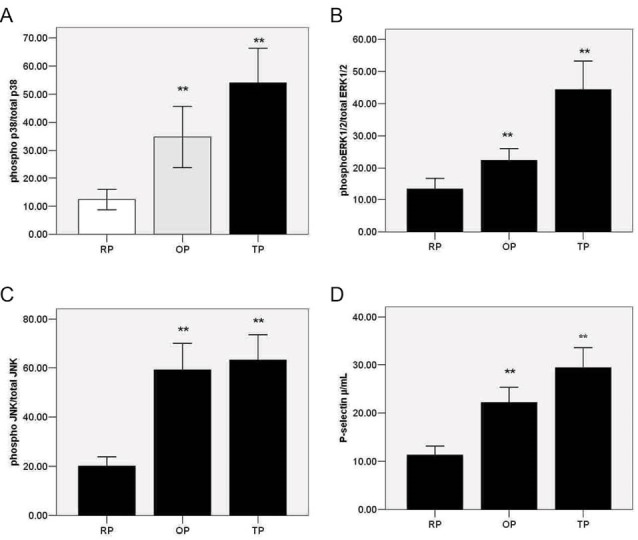
OxLDL and thrombin induce phosphorylation of p38, c-JNK and ERK1/2 in platelets. Whashed human platelets (5×108/ml) were incubated with 50 µg/ml oxLDL or 0.5 U/ml thrombin for 10 minutes and then lysed. The lysates were analyzed by solid phase sandwich Enzyme Linked-Immuno-Sorbent Assay (ELISA) for total p38, p-p38 (A), totalERK1/2, p-ERK1/2 (B), total JNK, p-JNK (C) and P–selectin expression (D). We showed all result by the ratio percentage of phosphorylated to total protein for normalizing the phospho-protein contents of cells (n=10 in all groups). RP, resting platelets; OP, oxidized-LDL treated platelets; TP, thrombin treated platelets. *P < .05, ** *P* <.005.

We measured phospho-p38 and total p38in the presence or absence of stimuli; thrombin or Cu^+2^-OxLDL and inhibitors; Se or SB 203580. The mean of total p38 in any groups was not statistically different from each other (*P *= .77). Exposure to OxLDL for 10 minutes significantly increased the phosphorylation of p-P38 in the OP group (*P* <.05) (Figure 1A). A Similar result was observed in the TP group (*P* < .05) (Figure 1B), but thrombin was the stronger stimulus than OxLDL (Figure 1).

**Figure 2:**
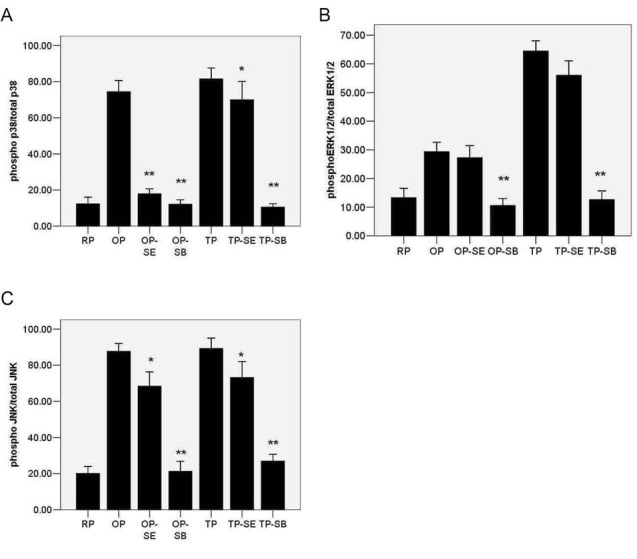
Effect of Selenium (Se) treatment on p38, ERK1/2 and JNK phosphorylation. Human platelets were incubated with 100 nmol/l Se for 48 hours, 5 × 103 nmol/l SB203580, 10 µM ERK kinase inhibitor PD980559 or, 400 nM JNK inhibitor SP6000125 for 30 minutes prior to incubation with 50 µg/ml ox LDL or 0.5U/m thrombin. Then the platelet lysates were analyzed by solid phase sandwich Enzyme Linked-Immuno-Sorbent Assay (ELISA) for totalp-38, p-p38 (A), totalERK1/2,P-ERK1/2 (B), total JNK, p-JNK(C). RP, resting platelets; OP, oxidized-LDL treated platelets; TP, thrombin treated platelets; SB, SB203580; PD, PD980559; SP, SP6000125. *P < .05, ** *P* <.005.

Se significantly decreased the OxLDL-induced p38 phosphorylation (*P* < .01) (Figure 2A). Thrombin-induced p-p38 also decreased significantly in the presence of Se (*P* < .05) (Figure 2B). We used a well-known p38 inhibitor called SB203580 to better elucidate the effect of Se on the p38 signaling pathway. Pretreatment of platelets with SB203580 (5 mmol/l) for 30 minutes and stimulation by OxLDL significantly decreased the OxLDL-induced (*P* < .01) and thrombin-induced (*P* < .01) p-p38 (Figure 2A and 2B).

### Inhibition of ERK1/2 Phosphorylation by Selenium

We assayed the effects of Se treatment on ERK1/2 phosphorylation in the OP and TP groups. The mean of total pERK1/2 in any group was not statistically different from each other (*P *= .92). Phosphorylation rate of ERK1/2 in thrombin-treated platelets was more than that of the platelets treated with OxLDL (*P* < .01). We observed a 4- to 5-fold increase of p-ERK/totalERK1/2 in TP (*P* < .01) (Figure 1B), and a 1.5-fold increase in OP (*P* < .05) (Figure 1A) compared with RP. A significant decrease inp-ERK1/2 was observed in OxLDL (*P* < .01) and thrombin-activated (*P* < .01) platelets using the specific ERK1/2 kinase inhibitor PD980559. P-ERK1/2 decreased in the presence of Se in the TP group (*P* < .05), unlike in the OP group (*P *= .48).

###  Inhibition of JNK Phosphorylation by Selenium

The results on p-JNK changes in the OP and TP groups in the presence or absence of Se were more similar to the p-p38 than p-ERK1/2 features. Significant decrease in the induced p-JNK by OxLDL (*P* < .05) or thrombin (*P* < .05) was observed. Expression of p-JNK decreased in the presence of Se in the OP group (*P* < .05), which was much less than that of the JNK inhibitor SP6000125 effect (*P* < .01).

### Inhibition of P-selectin by Selenium

**Figure 3:**
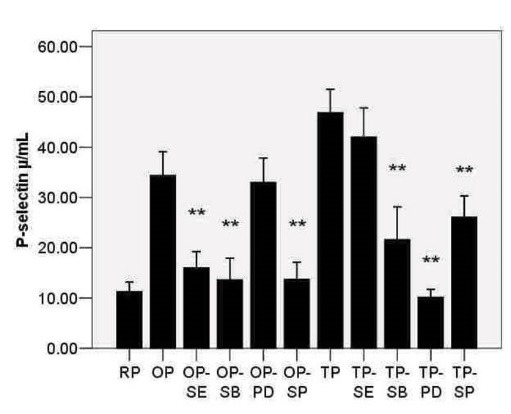
Comparisons of P-selectin levels in presence of Se, SB203580, PD980559 or SP6000125 after stimulation by cu2+-OxLDL or thrombin. Human platelets were incubated with 100 nmol/l Se, 5 mmol/l p38kinase inhibitor SB203580, 10 µM ERK kinase inhibitor PD980559 or 400 nM JNK inhibitor SP6000125 for 30 minutes prior to incubation with 50 μg/ml oxLDL or 0.5 U/m thrombin. The platelet lysates were then analyzed by solid phase sandwich Enzyme Linked-Immuno-Sorbent Assay for P-selectin expression n=10 in each group). P, resting platelets; OP, oxidized-LDL treated platelets; TP, thrombin treated platelets; SB, SB203580; PD, PD980559; SP, SP6000125. *P < .05, ** *P* <.005.

P-selectin expression in the RP, OP, and TP groups was also measured (Figure 3). The result showed that both of these stimuli increased the expression of P-selectin (*P* < .05; Figure 1D). Pretreatment with Se significantly decreased the expression of P-selectin induced by OxLDL (*P* < .05). Similarly, the inhibition of p-p38 by SB203580 or p-JNK by SP6000125can decrease P-selectin expression (*P* < .05) and (*P* < .05), respectively (Figure 3). However, ERK1/2 kinase inhibitor PD980559 did not significantly decrease the expression of P-selectin in OP (Figure 3).The effect of Se, PD980559, SB203580, and SP6000125 in TP was also considered. PD980559, SB203580, and SP6000125 can significantly decrease P-selectin levels (*P* < .05); however, Se could not reduce P-selectin in TP (*P* =.105) (Figure 3). A strong correlation was observed between p-p38/totalP38 (%) and pP-selectin in OP (r=0.65, *P* <.05) or TP (r=0.75,* P* < .05), p-JNK/total JNK(%) and pP-selectin in OP (r=0.72, *P* < .05) or TP, and between p-ERK1/2/total ERK1/2 (%)and pP-selectin in TP (r=0.79, *P* < .05). However, weak correlation was observed betweenp-ERK1/2/totalERK1/2(%) and pP-selectin in OP (r=0.24, *P* < .05) or between p-JNK/totalJNK (%) and pP-selectin in TP (r=0.20, *P* < .05) by Spearman correlation test.

## Discussion

This study aimed to explore by which mechanism Se can inhibit inflammatory events, particularly in platelets involved in the etiology of atherosclerosis.^10^ Platelets should be activated in order to act as inflammatory cells^11^ and experienced changes in size, shape, and proteome.^12^ P-selectin as a well-known marker of platelet activation, is associated with atherosclerosis by inducing platelet–endothelial and platelet–leukocyte adhesions.^13^ Platelet aggregation mean size strongly correlates with the P-selectin expression rate on the platelet surface.^14^ Moreover, an in vitro study showed that inhibition of P-selectin binding to its ligand by the anti–P-selectin antibodies G1 severely reduced the extent of platelet aggregation.^15^ The lectin domain of P-selectin is a means to attach the platelets together. These interactions stabilize the aggregates.^16,17^ Recent evidence has shown that MAPK is a key mediator in hemorrhagic damage of primary organs through the activation of pro inflammatory cytokines, such as interleukin (IL)-1β and tumor necrosis factor (TNF)-α.^16^ In this study, we confirmed the results from our previous study considering P-selectin expression in Cu^2+^ OxLDL or thrombin activated platelets and simultaneously over phosphorylation of wide range of MAPK pathway proteins, such as p38, ERK, and c-JNK.^12^

The innovative human health roles for Se in human health have been shown in recent studies.^18^ Genetic or ecologic causes, even the use of drugs, lead to a Se deficiency that has been reported in patients with different disorders including stroke,^19^ atherosclerosis, osteoarthritis^20^ and neuronal diseases such as depression, Alzheimer’s or amyotrophic lateral sclerosis,^21^ hypothyroidism,^22^ and diabetes. A Se supplement produced cytokine (eg, TNF-α and IL-1b)-induced P-selectin in diabetes mellitus along with increased oxidative stress.^23^ However, some studies have noticed the risk of overdose with Se. One study discovered that people who took 200 µg of Se a day (recommended dietary allowance for adults is 60 µg/day) were 50% more likely to develop type 2 diabetes.^24^ Another report indicated the correlation of Se overdosing with (squamous cell carcinoma).^25^ Regardless to advantage and disadvantage of selenium supplementation, several mechanisms have been assumed to mediate the effects of Se including antioxidant defense systems, selenoproteins expression pathways involved in oxidative metabolism, DNA intercalators, immune system regulation, kinase modulator, and gene expression.^26^ A relationship between the p38 signaling pathway and anti-oxidant system was also developed after hypoxia-oxygenation.^27^ The platelets had extremely high contents of Se, even under a micronutrient deficiency condition,^22^ which should be conserved and utilized correctly. After detecting of TLRs on the surface of platelets, these circulating particles were considered as inflammatory cells.^12^ Se probably reduced platelet inflammation by modulation of the MAPK pathways. We considered the P-selectin expression and phosphorylation rates of three main members of the MAPK family, MAPK p38, c-JNK, and ERK1/2, in the presence or absence of Se and their classic inhibitors, SB203580, SP6000125, and PD980559 in the activated platelets. Resultant data showed that OxLDL-induced p-P38 and p- JNK were significantly inhibited by Se, but not in p-ERK. Similar results were obtained by recent studies on endothelial cells.^28^ Our findings and these evidence collude for us to come up with the suggestion that the effect of Se supplementation on oxidative stress in platelets was partially concerns to the p38MAPK or c-JNK signaling pathways, but not to the ERK1/2. It should be mentioned that increase in p-ERK1/2 in OxLDL-treated platelets was slight and not significant. Of course dose/time dependent experiments may be elucidate more details. Despite of strong correlation between P-selectin and p-38 or p-JNK, we observed no change in levels of p-38 or p-JNK in presence of Se in thrombin activated platelets. The higher phosphorylation rate of ERK1/2 pathway in the thrombin-treated group, indicated that thrombin and OxLDL may use different pathways or mechanisms for platelet activation. Thrombin activates platelets mainly through protease-activated receptors 1 and 4 and the NF-κB signaling pathway.^12^ However, the OxLDL mechanism still remains elusive. Our experiments showed a similar pattern between Se and SB203580 or SP6000125 as classic anti-inflammatory drugs, on P-selectin expression in OxLDL treated platelets. Theses evidence confirms the involvement of Se in inflammatory process.

In the present study, the hypothesis that Se as a co-anti-inflammatory micronutrient can help to the prevention of OxLDL-induced platelets stimulation was confirmed.

## Limitations

The greatest limitation of the current study is that the platelets were treated in vitro and results might not apply to in vivo settings.

## Conclusion

We found that Se inhibits OxLDL-induced expression of P-selectin in platelets. This effect was at least partially mediated through the modulation of p38 or c-JNK signaling pathways. The results provided a new therapeutic insight for Se in atherosclerosis study.

## Ethical issues

This article was written based on a dataset of a PhD thesis registered in Tabriz University of Ethical issues. In addition, the protocol for the research project was approved by the ethics committee at TUMS (Tabriz University of Medical Sciences) in compliance with the Helsinki Declaration.

## Competing interests

The authors had no competing interests to declare in relation to this article.
